# Aggregation of the amyloid-β peptide (Aβ40) within condensates generated through liquid–liquid phase separation

**DOI:** 10.1038/s41598-024-72265-7

**Published:** 2024-09-30

**Authors:** Owen M. Morris, Zenon Toprakcioglu, Alexander Röntgen, Mariana Cali, Tuomas P. J. Knowles, Michele Vendruscolo

**Affiliations:** 1 Centre for Misfolding Diseases, Yusuf Hamied Department of Chemistry, University of Cambridge, Cambridge, CB2 1EW UK; 2 Cavendish Laboratory, Department of Physics, University of Cambridge, Cambridge, CB3 OHE UK

**Keywords:** Biochemistry, Biophysics

## Abstract

The deposition of the amyloid-β (Aβ) peptide into amyloid fibrils is a hallmark of Alzheimer’s disease. Recently, it has been reported that some proteins can aggregate and form amyloids through an intermediate pathway involving a liquid-like condensed phase. These observations prompted us to investigate the phase space of Aβ. We thus explored the ability of Aβ to undergo liquid–liquid phase separation, and the subsequent liquid-to-solid transition that takes place within the resulting condensates. Through the use of microfluidic approaches, we observed that the 40-residue form of Αβ (Αβ40) can undergo liquid–liquid phase separation, and that accessing a liquid-like intermediate state enables Αβ40 to self-assemble and aggregate into amyloid fibrils through this pathway. These results prompt further studies to investigate the possible role of Αβ liquid–liquid phase separation and its subsequent aggregation in the context of Alzheimer’s disease and more generally on neurodegenerative processes.

## Introduction

Alzheimer’s disease is a progressive neurodegenerative disorder, accounting for approximately two-thirds of all cases of dementia worldwide, with an estimated yearly societal cost exceeding $1 trillion^1,2^. The aggregation of the amyloid-β peptide (Aβ) into amyloid fibrils and amyloid plaques has long been associated with Alzheimer’s disease, although the exact nature of the association has not yet been fully clarified^3–5^. Aβ is an intrinsically disordered protein fragment that originates from the proteolytic cleavage of the amyloid precursor protein (APP)^3,4^. The reaction by which Aβ self-assembles from its monomeric soluble form into amyloid aggregates has been extensively studied in vitro^5–7^. Kinetic analysis has revealed that Aβ monomers initially interact with one another, in a process known as primary nucleation, to form oligomeric assemblies. These oligomeric assemblies then grow into amyloid fibrils through the addition of further Aβ monomers to their ends, in a process known as elongation. The amyloid fibrils, in turn, act as catalytic surfaces for the formation of new oligomeric assemblies. This surface-catalysed process is known as secondary nucleation, and it is the dominant mechanism for the proliferation of Aβ aggregates^5,6,8^.

Recently, however, an alternative pathway to amyloid formation has emerged, sometimes referred to as the condensation pathway^9^, whereby a protein aggregates into amyloid fibrils after undergoing a liquid–liquid phase separation process^8^. The liquid–liquid phase separation of proteins involves the separation of a homogeneous liquid phase into two coexisting liquid phases of different densities^10–14^. Within the high-density phase, nucleation processes, which are highly concentration dependent, are greatly accelerated, resulting in the rapid formation of amyloid aggregates. This condensation pathway to aggregation has been reported for proteins involved in neurodegenerative diseases, including α-synuclein, tau and FUS^15–20^. These observations, together with the suggestion that a large fraction of human proteins may undergo phase separation^21^, and the growing number of proteins for which this process has been reported in vivo^22^, are prompting the question of whether Aβ could also undergo amyloid aggregation from within liquid condensates. Recent evidence has shown that Aβ has the ability to undergo recruitment into other condensates^23^ while a kinetically trapped oligomeric state of this peptide can form condensates in the presence of surfactant molecules^24^. The question, however, as to whether monomeric Aβ can form condensates which then have the ability to mature and transition from a liquid state to a solid state still remains unanswered.

In this study, to probe the phase separation of Aβ, we used an in vitro microfluidic assay^16,25^ combined with fluorescence microscopy to observe the propensity of Aβ40 to undergo liquid–liquid phase separation. We observed that Aβ40, in the presence of claramine, a small molecule in the aminosterol class^25,26^ that has recently been reported to facilitate the liquid–liquid phase separation of α-synuclein^27^, had the ability to form a liquid-like condensate state. Moreover, using this microfluidic approach, we investigated the phase space of this system, monitoring various biophysical parameters such as droplet coalescence and Ostwald ripening. Finally, through the use of amyloid-specific fluorescent dyes, we observed a liquid-to-solid transition of Aβ40, resulting in the formation of Aβ40 amyloid fibrils within the dense liquid phase.

## Results and discussion

### Liquid–liquid phase separation of Aβ40

**Fig. 1:**
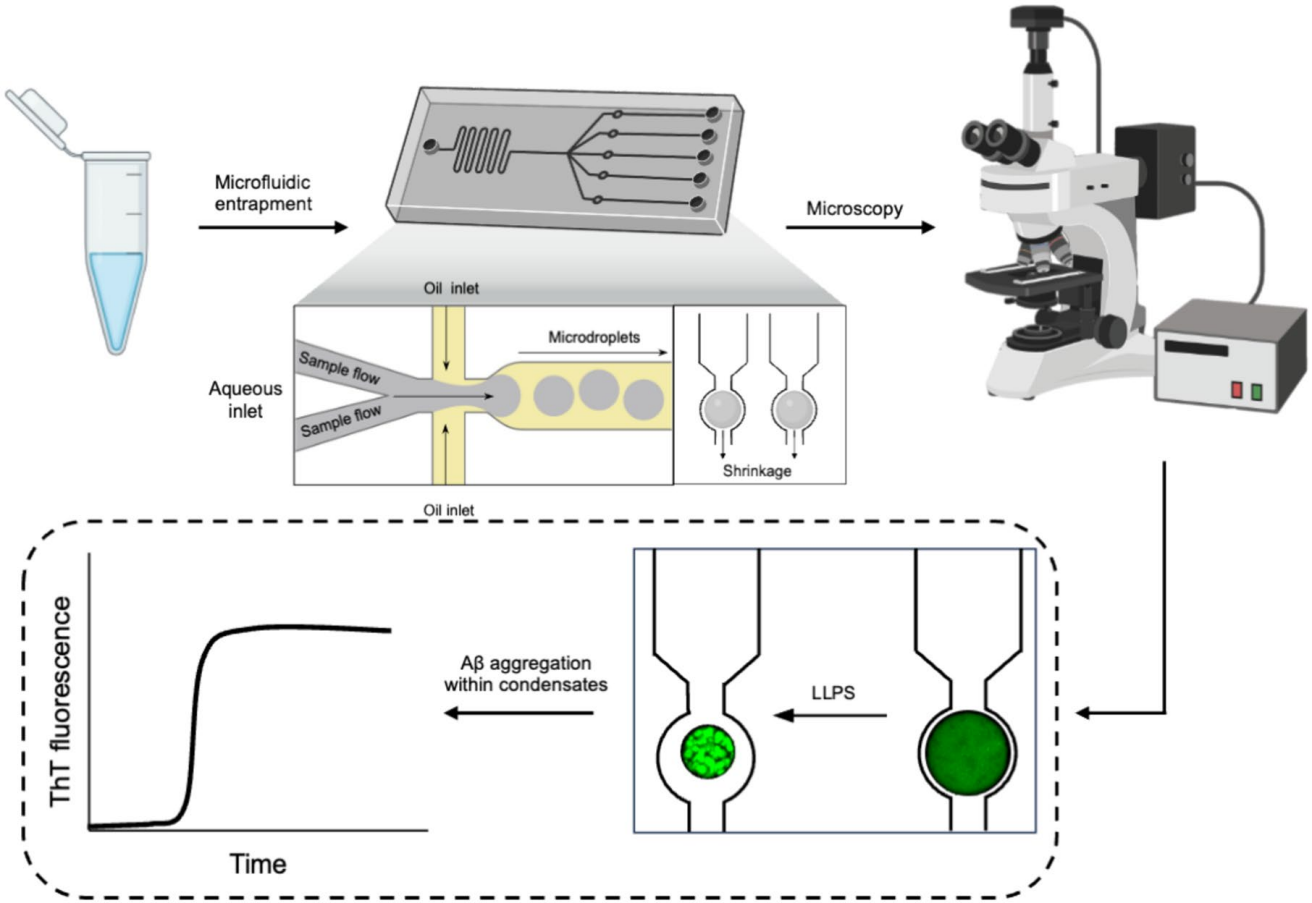
Schematic of the microfluidic strategy used to monitor liquid–liquid phase separation and subsequent aggregation of Aβ40 within the liquid-like condensates. An aqueous Aβ40 sample (3–5 μL) is initially injected into the microfluidic device. Following this, at the first junction, a solution containing the relevant stoichiometry of claramine was introduced to the Aβ40 sample. Aqueous microdroplets are generated at the second junction, where an oil phase intersects the flow of the aqueous solution containing Aβ40, PEG, and claramine. These microdroplets are subsequently entrapped within the device^38^. Fluorescence microscopy is then utilised to monitor the shrinkage of these microdroplets over time, which in turn induces the liquid–liquid phase separation of Aβ40. Time-lapse images of the microdroplets can then be analysed using Fiji^53^. The liquid–liquid phase separation and subsequent aggregation of Aβ40 can then be characterised. Depiction of the microfluidics device and fluorescence microscope have been adapted using BioRender (BioRender Premium, https://www.biorender.com/).

A general workflow for investigating the liquid–liquid phase separation of Aβ40 by using microfluidics is outlined in Fig. 1. With this workflow, we found that incubating Aβ40 with claramine in the presence of a macromolecular crowding agent such as polyethylene glycol (PEG), results in liquid–liquid phase separation and the generation of biomolecular condensates. PEG has been used a macromolecular crowding agent to induce phase separation for a variety of different systems, including the amyloidogenic proteins α-synuclein and tau^9,16,17,28^. Claramine, which is a synthetic analogue of the natural aminosterol trodusquemine, is a small cationic molecule consisting of a polyamine (spermine) moiety covalently bound to a cholestane sterol group. Aminosterols were first discovered when squalamine was isolated from stomach and liver extracts from *Squalus acanthias* and shown to have antimicrobial properties^29,30^. Recently, there has been a growing interest in the use of aminosterols as potential therapeutics in neurodegenerative diseases^31,32^. This interest has stemmed from studies reporting the ability of squalamine and related compounds to reduce the cytotoxicity of α-synuclein and Αβ oligomers^26,33–37^.

**Fig. 2:**
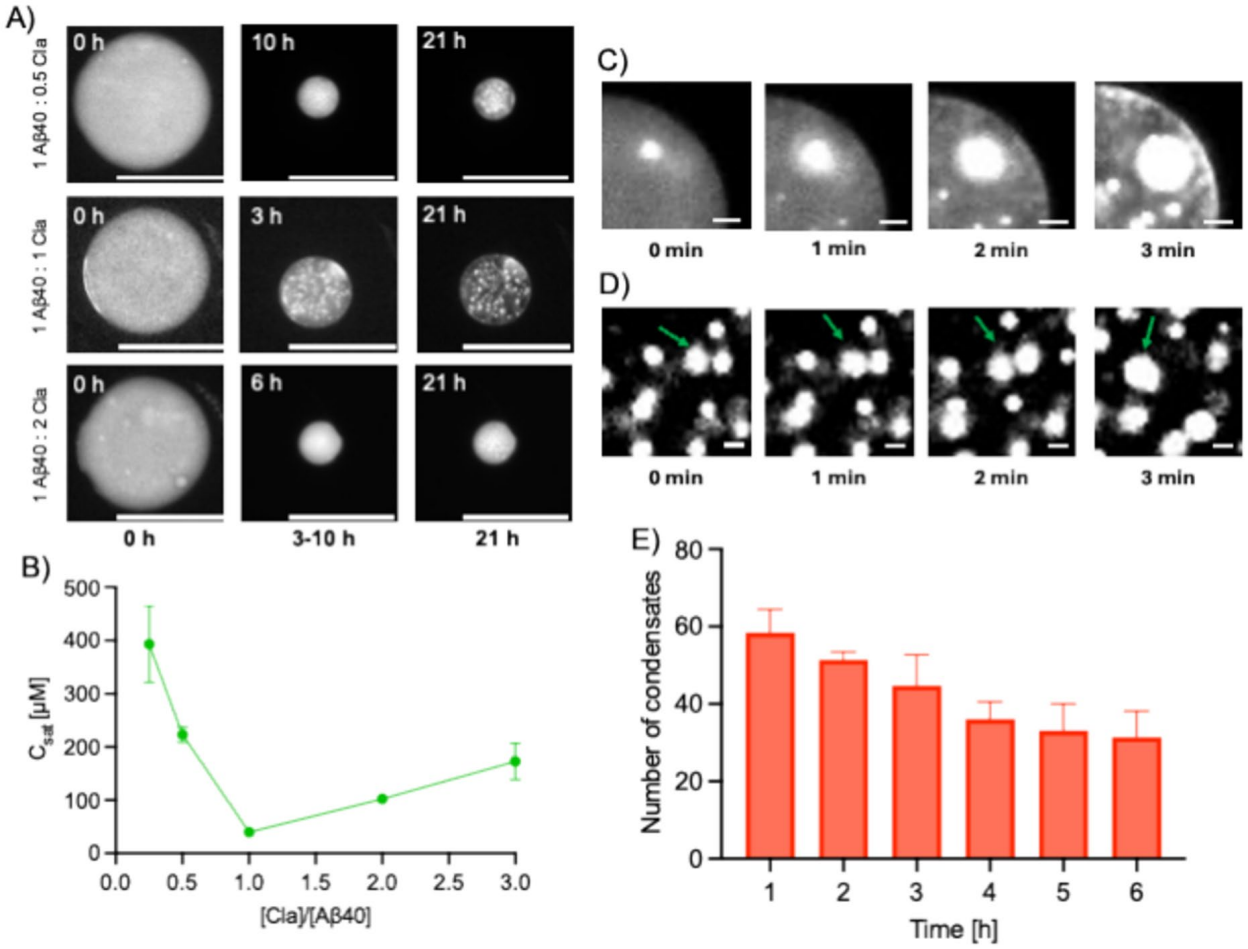
Formation of Aβ40 condensates through liquid–liquid phase separation. (**A**) Time-lapse fluorescence microscopy images of microdroplets with Aβ40 condensates forming within them as the individual microdroplets shrink. (**B**) Plot of the critical concentration at which liquid–liquid phase separation was observed (C_sat_) for Aβ40 as a function of the Aβ40:claramine ratio. Three data points were averaged for each C_sat_ value. Scale bar is 100 μm. (**C**) Fluorescence microscopy time-lapse images displaying Ostwald ripening of Aβ40 condensates. Scale bar is 10 μm. (**D**) Fluorescence microscopy time-lapse images displaying coalescence events occurring between Aβ40 condensates. The green arrows show the region at which the condensates coalesce. Scale bar is 5 μm. (**E**) Bar chart showing the decrease in the total number of condensates over time for a 1:1 ratio of Aβ40 to claramine. Analysed condensates were selected based on those being in-focus within the fluorescence microscopy images. Data are shown as mean ± SD of n = 3.

To investigate the propensity of Aβ40 to undergo liquid–liquid phase separation, Aβ40 samples together with 5% PEG and with varying amounts of claramine were injected into a microfluidic device^38^ (see “Materials and methods”). This approach involves the use of an array of microfluidic traps whereby aqueous sample-containing microdroplets are confined and studied over time (Fig. 1). These water-in-oil droplets are stabilised by surfactant molecules that localise at the water–oil interface. In this procedure, water molecules can escape from the droplet and partition into the oil phase. This is possible as water molecules can be encapsulated within inverse micelles, resulting in the diffusion of the aqueous phase through the highly porous PDMS, which eventually leads to droplet shrinkage. This process has been previously observed for trapped droplets in PDMS chambers^39^. Moreover, to avoid microdroplet coalescence and surface effects, we used a fluorinated oil^16^. This oil was specifically chosen as it has been shown to minimise any interactions between biomolecules and claramine, so our observations could be considered to be surface independent. It is important to note that in the absence of a microfluidic setup where we encapsulated Aβ40 within an aqueous droplet to investigate its propensity to phase separate on a glass slide, we observed that the Aβ40 aggregated almost instantaneously, before being able to access the condensed state. That is because Aβ has already been shown to be extremely sensitive to the air interface^40,41^, and the aggregation pathway is significantly induced once Aβ is exposed to an air interface. Therefore, a conventional drop-cast experiment could not be conducted to monitor the phase separation of Aβ40. However, using a water-in-oil droplet, one circumvents the air interface and thus, by encapsulating Aβ40 within aqueous-based microdroplets, we could study the liquid–liquid phase separation of Aβ40. Fluorescence microscopy was utilised to monitor the time-dependent shrinkage of these microdroplets, which in turn induced the liquid–liquid phase separation by enhancing the concentration of Aβ40, claramine, and PEG within the microfluidic droplet (Supplementary Table 1). This procedure allowed us to observe Aβ40 condensates. The liquid–liquid phase separation of Aβ40 was monitored within the microdroplets for various stoichiometries of Aβ40:claramine (Fig. 2A), using a small proportion of AF488-labeled Aβ40 (see “Materials and methods”). These individual microdroplets containing Aβ40 condensates were also visualised using confocal microscopy (Supplementary Fig. 1). A 1:1 ratio of Aβ40:claramine was found to be most effective for promoting liquid–liquid phase separation. This finding was confirmed by calculating the saturation concentration (C_sat_), which is the concentration of Aβ40 required to generate biomolecular condensates^8^ (Fig. 2B). The electrostatic interactions between the negatively charged Aβ40 and the cationic polyamine moiety of claramine (spermine) are likely responsible for driving the process of liquid–liquid phase separation. A previous report identified the electrostatic interactions between spermine (a component of trodusquemine) and Aβ42 as the primary driver in accelerating the aggregation of Aβ42, with the most potent effect observed at a 1:1 ratio^36^. Therefore, a 1:1 stoichiometry of Aβ40:claramine may provide sufficient association to promote the phase separation under the conditions tested. Moreover, in order to establish that claramine is necessary for the liquid–liquid phase separation of Aβ40, negative control experiments were conducted. We thus determined that only in the presence of both PEG and claramine Aβ40 undergoes phase separation. Moreover, we found that a solution containing just PEG with claramine did not phase separate (Supplementary Fig. 2a). All the negative control results are summarised in Supplementary Fig. 2.

Monitoring the Aβ40 condensates indicated typical liquid-like behaviour, as indicated by the physical processes that drive the growth of liquid droplets and the spherical appearance of condensates. Aβ40 condensates were observed to undergo Ostwald ripening, where larger coacervates grow rapidly at the expense of smaller coacervates (Fig. 2C). Furthermore, the coalescence of Aβ40 condensates was also observed, as condensates of increasingly larger sizes were produced (Fig. 2D and Supplementary Fig. 3A, B). The green arrows in Fig. 2D indicate the region at which the condensates coalesce over time. Both processes discussed above can be seen, along with the maturation of Aβ40 condensates when monitoring the microdroplets within the microfluidic device over time (Supplementary Videos 1 and 2). Furthermore, a liquid-like behaviour is corroborated by observing the number of Aβ40 condensates within an individual microdroplet. As the microdroplet shrinks and the critical concentration is reached at which liquid–liquid phase separation is induced, a decrease in the total number of condensates is detected due to coalescence events (Fig. 2E). This indicates that at this point, the condensates are still liquid as they have the ability to coalesce. Eventually, the number of Aβ40 condensates reaches a plateau, as the individual condensates mature and lose their liquid properties as they become more gel-like and exhibit solid-like behaviour. This is characteristic of phase-separated systems, and has been reported for different proteins including α-synuclein^9,15,16,42^, FUS^19,43^, and TDP-43^44,45^.

### Aggregation of Aβ40 within liquid condensates

Next, we sought to investigate the propensity of Aβ40 to aggregate within the liquid condensates. To study the self-assembly of Αβ40 into the amyloid state, we added the amyloid binding dye thioflavin T (ThT). ThT is a fluorescent dye that has the ability to bind to the cross-β core of amyloid fibrils, and in doing so it increases its quantum yield, thereby exhibiting an increased fluorescence intensity at 490 nm^46,47^. Additionally, the use of labelled Aβ40 was substituted for wild-type Aβ40 to eliminate any crosstalk between the emission intensity of AF488-Aβ40 and the emission intensity of ThT.

**Fig. 3:**
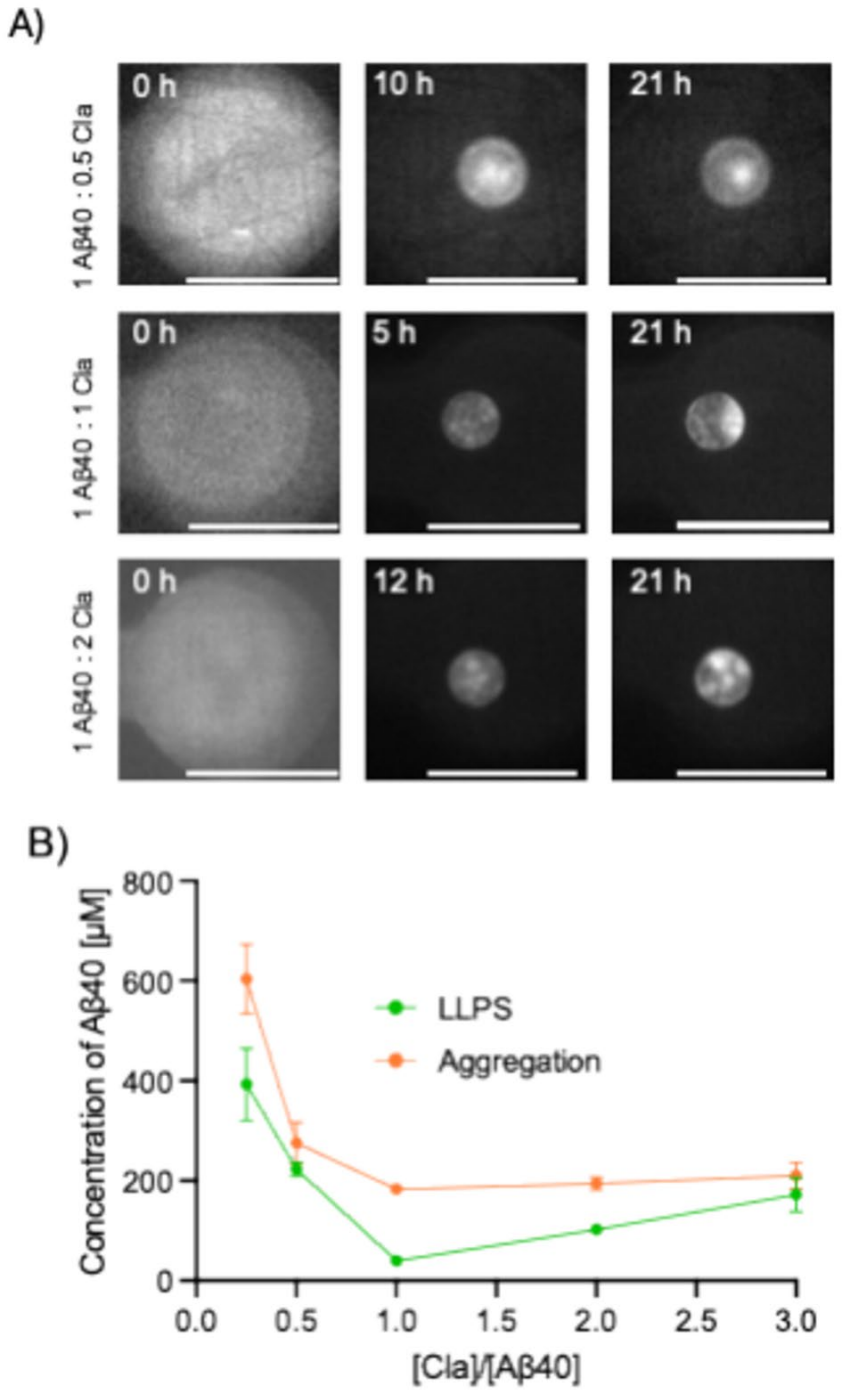
Aggregation of Aβ40 within liquid condensates. (**A**) Time-lapse fluorescence microscopy images of Aβ40 undergoing aggregation within condensates. Scale bars are 100 μm. (**B**) Phase diagram of Aβ40 concentration at which phase separation and subsequent aggregation takes place for varying Aβ40:claramine ratios. The green line displays the concentration at which Aβ40 undergoes liquid–liquid phase separation at various Aβ40:claramine stoichiometries. The orange line displays the concentration of Aβ40 at which the initial stages of aggregation are observed within the condensed liquid phase. Data are shown as mean of n = 3.

Microfluidic experiments involving 7 μΜ of Aβ40 in combination with a 0.25–3 stoichiometric ratio of claramine were conducted in the presence of 5% PEG and 20 μM ThT. As the individual microdroplets within the microfluidic device shrank over time, liquid–liquid phase separation was once again observed for Aβ40 for all stoichiometries of claramine tested (Fig. 3A). We note that as the experiment proceeds, the rate of droplet shrinkage is greatly reduced. This process likely occurs because of two complementary processes. Firstly, when the water escapes from the aqueous droplets into the oil phase, it eventually enters the porous PDMS. If enough water escapes, then the PDMS becomes saturated, leading to a decrease in the rate of evaporation. Additionally, when liquid–liquid phase separation occurs, a protein is able to retain water molecules^48^. Subsequently, at much later time points in the experiment (i.e., when we observe the liquid–liquid phase separation and subsequent aggregation) the rate of droplet shrinks at an extremely low rate. We therefore assume that the rate of amyloid formation is far greater than the rate of droplet shrinkage.

We then observed that Aβ40 condensates underwent a liquid-to-solid transition, as indicated by an increase in the ThT intensity within Aβ40 condensates. By monitoring this process with fluorescence microscopy, we calculated the critical concentration of Aβ40 within each microdroplet required to initiate the aggregation of Aβ40 within the condensates (Fig. 3B). Similar to the findings described above, a 1:1 Aβ40:claramine ratio was optimal for promoting Aβ amyloid formation within the liquid condensates. At lower claramine stoichiometries, we observed that a sharp increase in the critical concentration of Aβ40 was required to initiate the aggregation process, whereas at stoichiometric ratios beyond 1:1, a gradual increase in Aβ40 critical concentration was determined (Fig. 3B).

Combining the results for the C_sat_ of liquid–liquid phase separation of Aβ40 (Fig. 2B) and for the critical concentration needed to initiate the liquid-to-solid phase transition, (Supplementary Fig. 4) generates a phase boundary of Aβ40 in the presence of claramine (Fig. 3B) for both regimes. Thus, the phase boundary at which the liquid–liquid phase separation of Aβ40 was observed in the presence of claramine was determined (green curve in Fig. 3B), as well as the phase boundary at which the Aβ40 condensates aggregate to amyloid fibrils (orange curve in Fig. 3B). Figure 3B, therefore, shows the boundary details of the concentrations of Aβ40 required to induce its liquid–liquid phase separation and form coacervates at each stoichiometry (green curve in Fig. 3B), along with the Aβ40 concentration at which the aggregation within the coacervates is initiated (orange curve in Fig. 3B).

### Kinetic analysis of Aβ40 aggregation within liquid condensates

**Fig. 4:**
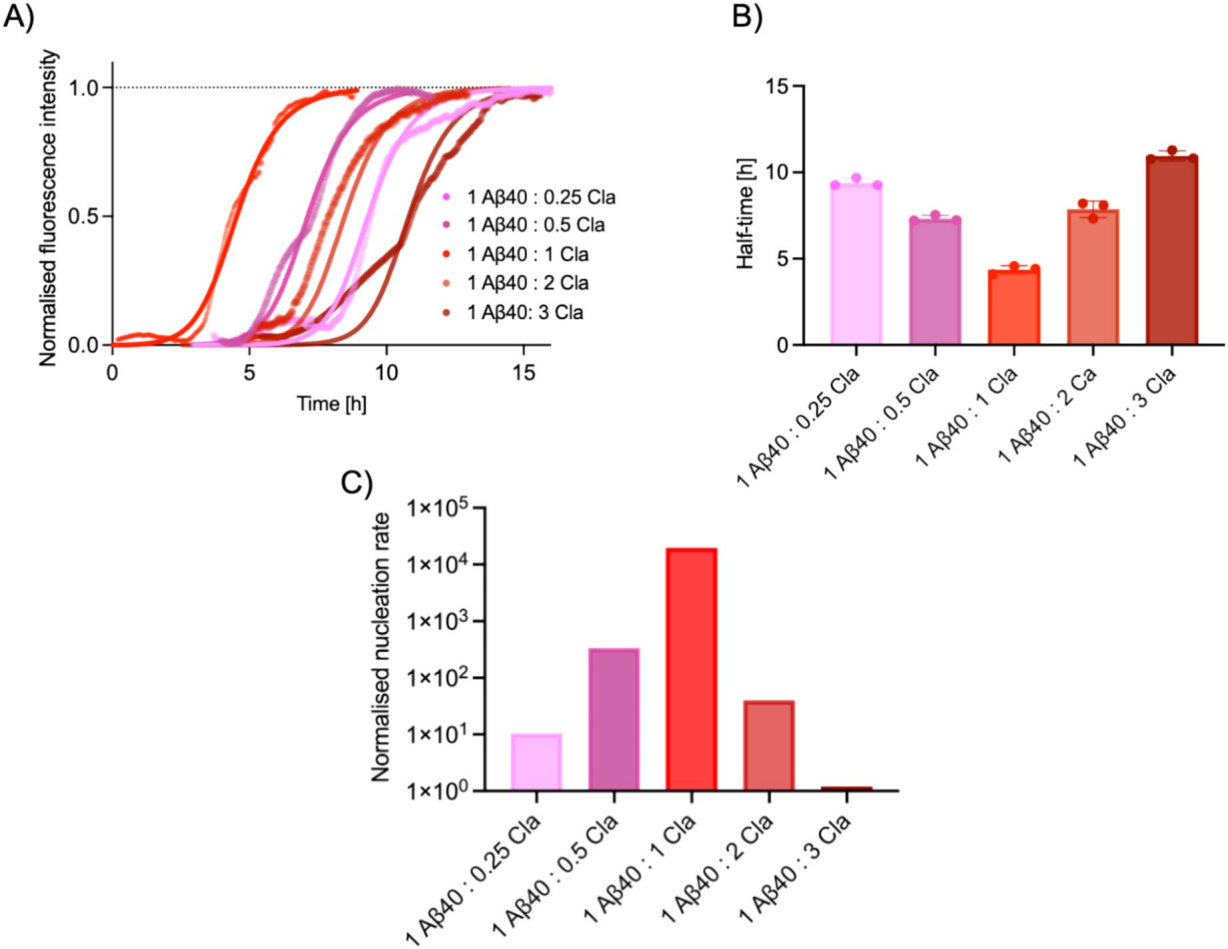
Aggregation kinetics of Aβ40 within liquid condensates. (**A**) Normalised kinetic traces for Aβ40 aggregation within liquid condensates at various Aβ40:claramine stoichiometries. The solid lines represent the fits to the kinetic data, which was performed using AmyloFit^49^. Data are shown as mean ± SD of n = 3 (**A**–**C**). The legend indicates the colour code used throughout the figure for the Aβ40:claramine stoichiometry. (**B**) Bar chart of the half-time of Aβ40 aggregation within liquid condensates for various Aβ40:claramine stoichiometries. (**C**). Bar chart of the normalised nucleation rate as a function of the Aβ40:claramine stoichiometry.

Further analysis of these microfluidic experiments involving Aβ40 and ThT yielded a series of kinetic aggregation curves for each Aβ40:claramine ratio (Fig. 4A). These individual kinetic traces follow a characteristic sigmoidal pattern, whereby a rapid exponential growth phase is preceded by a lag phase and followed by a plateau phase^5,8^. The raw kinetic traces for each Aβ40:claramine ratio were then normalised (Fig. 4A). The normalised kinetic curves support the conclusion that a 1:1 ratio of Aβ40 to claramine aggregates within the condensed liquid state more readily than other stoichiometries. This finding was also reinforced by extracting the half-time of aggregation, which is the time taken for the fibrillar mass to reach half of its plateau value (Fig. 4B).

To elucidate the microscopic processes that dominate the macroscopic aggregation behaviour, a global fitting platform, AmyloFit, which compares the integrated rate laws to experimental kinetic data^49^, was used. By employing a chemical kinetics framework, we were thus able to elucidate the rate constants of the microscopic steps involved in this aggregation process through the condensation pathway^16^. These microscopic steps include primary nucleation (k_n_), elongation (k_+_) surface-catalysed secondary nucleation (k_2_). The reaction order of the primary process is denoted by (n_c_), while the reaction order of the secondary process in the pathway is denoted by (n_2_). Importantly, only two key rate parameters control the time course of aggregation. These are the combinations of rate constants k_+_k_n_ and k_+_k_2_, which describe aggregate proliferation through primary and secondary nucleation, respectively^40,50^.

We first obtained the rate parameters for the 1:1 Aβ40:claramine ratio, and then the rate parameter for secondary nucleation, k_+_k_2_, was kept constant throughout the analysis at 3.61 × 10^15^ M^−3^ h^−2^. The rate parameter for primary nucleation, k_+_k_n_, for each system was then obtained (Fig. 4C). The concentration used for Aβ40 in this fitting model was that obtained when calculating the concentration within the condensed phase, and not the concentration of Aβ40 at the initial state of the system. We found that small differences in the Aβ40:claramine ratio can greatly modulate the rate parameter for primary nucleation, in some cases affecting it by up to 5 orders of magnitude, as can be seen in the plot of the normalised rate constant as a function of the Aβ40:claramine ratio in Fig. 4C**.** Furthermore, a non-monotonic behaviour between the nucleation rate and the Aβ40:claramine ratio was observed, with the maximum rate obtained when the Aβ40:claramine ratio was 1. These data suggest the presence of two regimes. In the low Aβ40:claramine ratio, the primary nucleation rate is supressed (Fig. 4C). This implies that a critical amount of claramine is needed to induce condensate formation and subsequent aggregation within the condensates. In the high Aβ40:claramine ratio regime, the primary nucleation rate is supressed. This suggests that although claramine can induce phase separation and aggregation, if it is in excess, it can also interfere with these processes. We note that claramine has already been shown to stabilise the condensed phase and affect the primary nucleation rate of another amyloidogenic protein, α-synuclein^27^.

To identify the amyloid aggregates formed within liquid condensates, transmission electron microscopy (TEM) was utilised to confirm whether Aβ40 formed fibrils for all stoichiometric ratios. Amyloid fibrils were recovered from condensates and fibrillar aggregates were in fact observed for all conditions tested (Supplementary Fig. 5).

## Conclusions

**Fig. 5:**
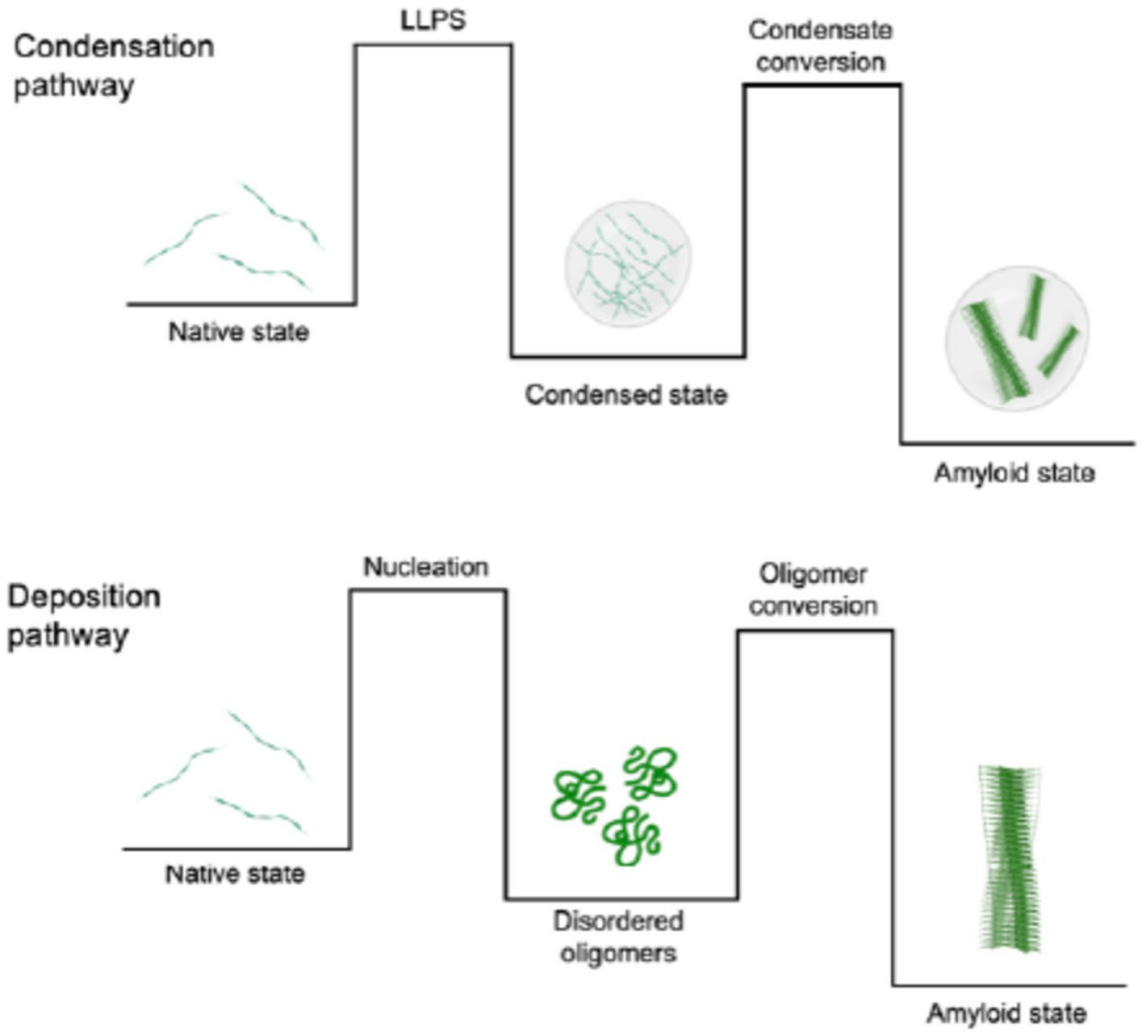
Schematic representation of the condensation and deposition pathways involving the aggregation of Aβ40. The conversion between the native and amyloid states along the condensation pathway takes place through an intermediate condensed state. Along the deposition pathway, the conversion takes place through the formation of disordered oligomers.

We reported that Aβ40 can undergo liquid–liquid phase separation under suitable conditions. Using a microfluidic-based approach coupled with fluorescence microscopy, we further showed that Aβ40 condensates can mature with time, resulting in the formation of amyloid aggregates. These results show that Aβ40 can aggregate through the condensation pathway, as summarised in Fig. 5. In this pathway, Aβ40 first populates a condensed state by undergoing liquid–liquid phase separation. The resulting condensates then readily convert into amyloid fibrils due to the high concentration of protein within the condensates, which greatly enhances primary nucleation. We anticipate that the condensation pathway of Aβ will be further investigated as an alternative mechanism for Aβ to aggregate, given its potential significance in amyloid formation and, ultimately, in the development of Alzheimer’s disease.

## Materials and methods

### Purification and fluorescent labelling of Aβ40

Human wild-type Aβ40 and its cysteine-bearing variant (insertion at position 2) were expressed in *E. coli* BL21 Gold (DE3) competent cells, transformed with a pT7 plasmid encoding the relevant Aβ40 variant. The Aβ40 variants were subsequently purified in 50 mM Tris–HCl pH 7.4 using a previously established protocol to achieve consistent properties^51,52^. All Aβ40 was aliquoted, flash-frozen in liquid nitrogen, lyophilised, and stored at -80 °C. Lyophilised Aβ40 was redissolved in 50 mM Tris–HCl pH 7.4 on ice at a stock concentration of (50 μM) before each experiment.

The cysteine-bearing Aβ40 variant was redissolved in 50 mM NaPi pH 7.5 and labelled with an excess of Alexa Fluor™ 488 C_5_ Maleimide (AF488; Invitrogen Life Technologies) and incubated at room temperature for 2 h. Successful labelling was confirmed by mass spectrometry (Supplementary Fig. 6). Labelled Aβ40 was separated from unbound dye and Aβ40 dimers by size exclusion chromatography (23 mL Superdex 75 10/300 GL column) using a flow rate of 0.7 mL min^−1^ 50 mM Tris–HCl pH 7.0. The concentration of labelled Aβ40 was determined from the Beer-Lambert law by measuring the UV–Vis absorbance at 495 nm using an extinction coefficient ε = 73,000 M^−1^ cm^−1^ for AF488.

### Microfluidic liquid–liquid phase separation assay

*Liquid–liquid phase separation assay composition.* 7 μM of Aβ40 (10% AF488- labelled) was combined with 5% (w/w) polyethylene glycol (PEG) (10 kDa, Thermo Fisher Scientific) in 50 mM Tris–HCl pH 7.4 and varying stoichiometries of claramine (Sigma-Aldrich). Aqueous microdroplets consisting of a varying mixture of PEG/claramine and peptide were generated and confined in the microfluidic trapping device for subsequent imaging^38^.

#### Fabrication of microfluidic devices

The fabrication process of the microfluidics was taken from a previously established protocol^38^. A soft photolithographic process was used to fabricate the master through which microfluidic devices were made. A 50 μm photoresist (SU-8, 3050, MicroChem) was spin-coated onto a silicon wafer. This was soft baked at 95 °C for 3 min. A film mask was placed on the wafer and system was exposed to UV light to induce polymerization. The wafer was then baked at 95 °C for 30 min. Finally, the master was placed into a solution of propylene glycol methyl ether acetate (PGMEA, Sigma-Aldrich), which helped in the development process. Elastomer polydimethylsiloxane (PDMS) with curing agent (Sylgard 184, DowCorning, Midland, MI) was mixed at a ratio of 10:1 to fabricate he devices. This mixture was then incubated at 65 °C and cured for a total of 3 h. Once solidified, the PDMS was peeled off the master, and holes were punched into the PDMS, which acted as inlets and outlets. Finally, the PDMS slab was bound to a glass slide by treatment with a plasma bonder (Diener Electronic, Ebhausen, Germany).

#### Formation and confinement of microdroplets

Syringe pumps (neMESYS, Cetoni, Korbussen, Germany) were used to control the flow rates within the microchannels. As outlined in Fig. 1, the Aβ40 solution was introduced to the device, followed by an aqueous claramine solution at the first junction. At the second junction, the oil phase, which consisted of fluorinated oil (Fluorinert FC-40, Sigma-Aldrich) and 2% w/w fluorosurfactant (RAN biotechnologies) intersected the aqueous phase, which resulted in water-in-oil microdroplets being formed. Following microdroplet generation, microdroplets were entrapped within the microfluidic device. Briefly, microdroplets were directed towards an array of traps whereby once a microdroplet is driven within the microfluidic confinement, it is unable to escape unless a pressure Is applied from the outlet. Microdroplets were then incubated at room temperature to allow for shrinkage. This resulted in an increase of the local concentration of Aβ40 and PEG within the microdroplets which led to Aβ40 phase separation (Supplementary Fig. 5). The water-in-oil microdroplets and the Aβ40 phase separation was monitored using fluorescence microscopy (Bright-field and GFP-channel, 488 nm).

#### Calculation of Aβ40 concentration during phase separation

The concentration of Aβ40 required to induce liquid–liquid phase separation was obtained by calculating the ratio of the microdroplet volume following trapping and at the point of phase separation, i.e. the point at which condensates start appearing within the water-in-oil microdroplet^16^. Through this analysis, the Aβ40 concentration at the point of phase separation could be determined.

### Aβ40 aggregation assay within liquid condensates

7 μΜ Aβ40 was incubated with 5% (w/w) PEG (10 kDa, Thermo Fisher Scientific), 20 μM ThT (Sigma-Aldrich) and varying stoichiometries of claramine in 50 mM Tris–HCl pH 7.4. Approximately 5 μL of the assay mixture was injected into the microfluidics device, where microdroplets were entrapped and imaged at 490 nm using fluorescence microscopy. Images were processed using Fiji, where individual microdroplets were analysed.

### Confocal microscopy

Approximately 5 μL of Aβ40 sample was injected into the microfluidic device using the method outlined in the “Formation and confinement of microdroplets” section and imaged using a Leica TCS SP8 inverted confocal microscope using a 20× objective (Leica microsystems). The excitation wavelength was set at 490 nm. Images were processed using Fiji.

### Transmission electron microscopy (TEM)

TEM was performed using a Talos F200X G2 transmission electron microscope operating at 80–200 kV. TEM images were acquired using a Ceta 16 M CMOS camera. TEM grids (continuous carbon film on 300 mesh Copper grid) were glow discharged using Quorim Technologies GloQube instrument at a current of 25 mA for 60 s. 5 μL of sample was then deposited for 30 s before being blotted. The grid was then washed 3 times with 5 μL of de-ionised water. 5 μL of a 2% (w/v) uranyl acetate solution was added to the grid (for a total of 40 s) in order to negatively stain the sample. The samples were then imaged.

### AmyloFit kinetic analysis

All kinetic data obtained were analysed using AmyloFit^49^. All datasets were initially normalised prior to upload. The half-times were calculated from the time at which the fibrillar mass reached half its maximum value, i.e., the time at which the normalised fluorescence intensity reached 0.5 (this is explained in more detail below). Each experiment was repeated three times and averaged before fitting. From the data, a secondary nucleation dominated model was found to be the best model to use. The fitting was initially performed on the 1:1 Aβ40:claramine ratio and subsequently, all parameters other than the k_+_k_n_ were kept constant. All kinetic parameters obtained are plotted in Fig. 4C.

### Normalisation of kinetic data

In order to fit the data using AmyloFit, and obtain the kinetic parameters and nucleation rates, we first had to normalise the data. Importantly, the aggregation kinetics were monitored from the point at which condensate formation was observed. Specifically, the half-time values shown in Fig. 4 were calculated as the time from the onset of condensate formation to the point at which the fibrillar mass reached half its maximum value.

## Supplementary Information

Supplementary Information 1.

Supplementary Video 1.

Supplementary Video 2.

## Data Availability

All data generated or analysed during this study are included in this published article and its supplementary information files.
