# Supplementary material for: Aggregation of the amyloid-β peptide (Aβ40) within condensates generated through liquid–liquid phase separation
Source: Sci Rep. 2024 Sep 30;14:22633. doi: 10.1038/s41598-024-72265-7 (PMC11442885; doi:10.1038/s41598-024-72265-7)
Supplement: Supplementary file 1 — Supplementary Information 1. [file 41598_2024_72265_MOESM1_ESM.docx]

**SUPPORTING INFORMATION**

**Aggregation of the Amyloid-β Peptide (Aβ40) within Condensates Generated through Liquid-Liquid Phase Separation**

Owen M. Morris^1,+^, Zenon Toprakcioglu^1,+^, Alexander Röntgen^1^, Mariana Cali^1^,

Tuomas P. J. Knowles^1,2^, and Michele Vendruscolo^1^*

^1^*Centre for Misfolding Diseases, Yusuf Hamied Department of Chemistry,*

*University of Cambridge, Cambridge CB2 1EW, UK*

^2^*Cavendish Laboratory, Department of Physics,*

*University of Cambridge, Cambridge CB3 OHE, UK*

*+ Equal contributions*

** Correspondence to: mv245@cam.ac.uk***Supplementary Video 1:** Time evolution of a condensate using fluorescence microscopy. The video shows the Ostwald ripening and coalescence of Aβ40 condensates within a microdroplet. 1:1 ratio of Aβ40:claramine.

**Supplementary Video 2:** Time evolution of a condensate using confocal microscopy. The video shows the Ostwald ripening and coalescence of Aβ40 condensates within a microdroplet. 1:1 ratio of Aβ40:claramine.

**
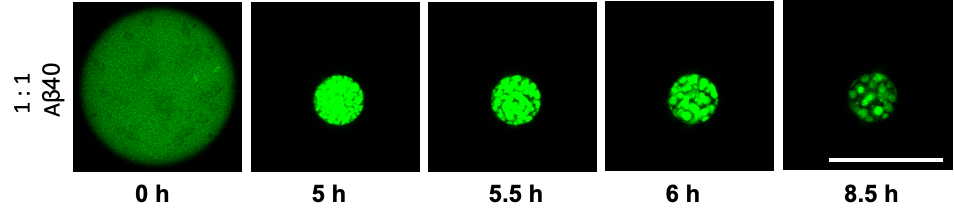
**

**Supplementary Figure 1.** **Time evolution of an individual microdroplet displaying the liquid-liquid phase separation of Aβ40.** Images were acquired by confocal microscopy using Alexa Fluor 488. Scale bar = 100 μm.

**Supplementary Figure 2. Negative controls for the phase separation of Aβ40**. **(A)** Fluorescence microscopy images of the time evolution of an individual microdroplet of 7 μM claramine in 50 mM TRIS-HCl at pH 7.4 in the presence of 5% PEG and in the absence of Aβ40. Due to the absence of the fluorescently-labelled Aβ40, the microdroplet was imaged using bright-field microscopy. The granular structures observed in the images are not condensates, but artefacts on the PDMS, as indicated by the granular structures outside of the droplet, and indeed even outside of the device itself. **(B)** Fluorescence microscopy images of the time evolution of an individual microdroplet of 7 μM Aβ40 incubated with 7 μM claramine in the absence of PEG. **(C)** Fluorescence microscopy images of the time evolution of an individual microdroplet of 7 μM Aβ40 incubated with 5% PEG in the absence of claramine. No phase separation was observed for any control. This indicates that Aβ40, claramine, and 5% PEG are all essential for inducing phase separation of Aβ40. Scale bar = 100 μm.


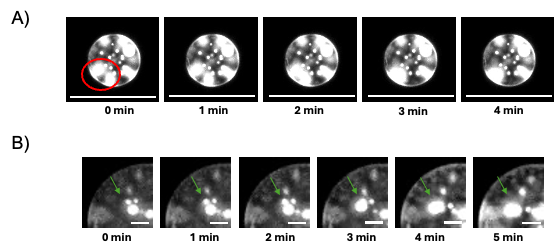


**Supplementary Figure 3. Coalescence of Aβ40 condensates.** Fluorescence imaging of two independent coalescence events occurring between Aβ40 condensates. Images were taken from the time evolution of two independent microdroplets at a 1:1 Aβ:claramine stoichiometry. Scale bar: 100 μm **(A)**, and 10 μm **(B)**.

**Supplementary Figure 4. Critical concentration of Aβ40 at which the aggregation within condensates was observed.** Data are shown as mean + SD of n=3.


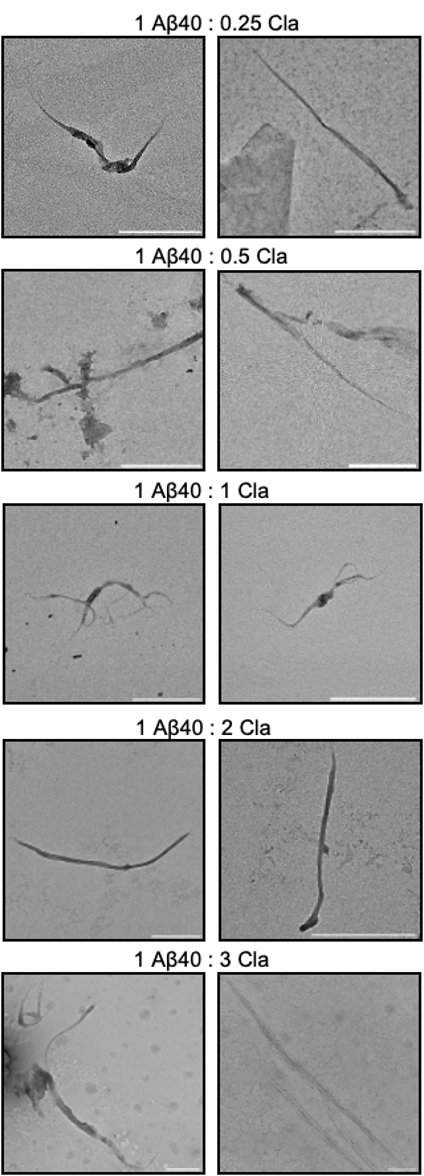


**Supplementary Figure 5. Transmission electron microscopy (TEM) images of Aβ40 fibrils**. Fibrillar aggregates of Aβ40 were imaged in the presence of various stoichiometries of claramine. Two micrographs are displayed for each condition. Scale bar shown is 1 μm for all images.


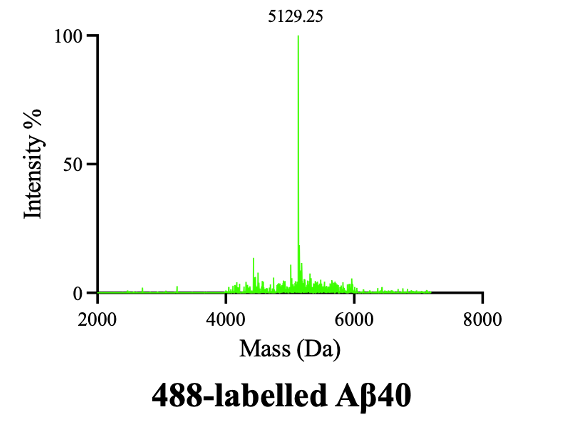


**Supplementary Figure 6. Mass spectrum of Aβ40 conjugated with Alexa Fluor 488.**


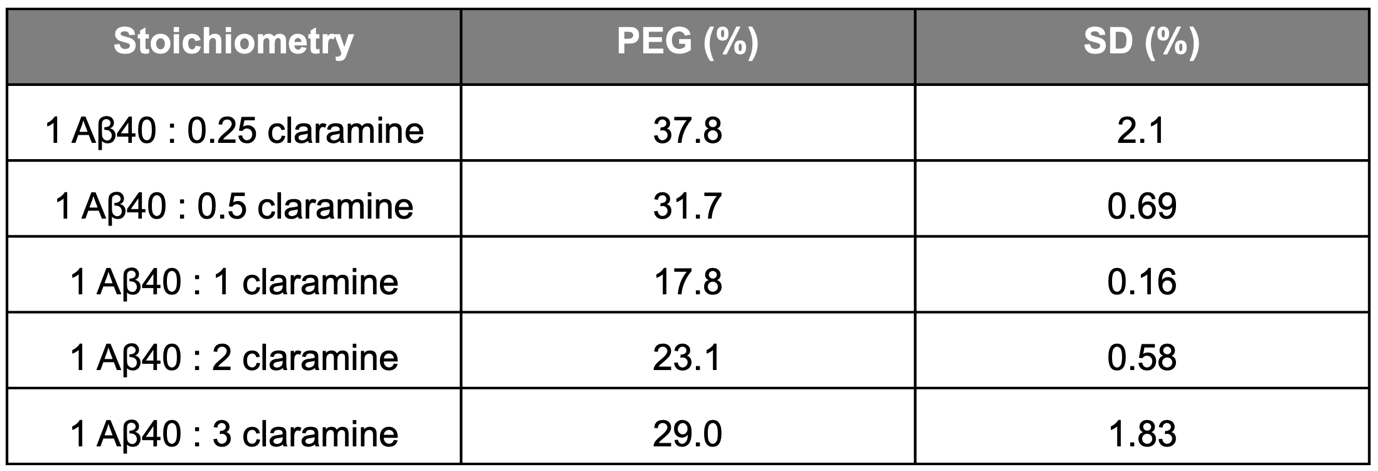


**Supplementary Table 1.** Table displaying the relative percentage of PEG within the microfluidic droplets at the point of phase separation. Standard deviation shown as a percentage. n=6.
